# High Ki67 expression, HER2 overexpression, and low progesterone receptor levels in high-grade DCIS: significant associations with clinical practice implications

**DOI:** 10.3389/fonc.2025.1467664

**Published:** 2025-01-28

**Authors:** Hossein Schandiz, Lorant Farkas, Daehoon Park, Yan Liu, Solveig N. Andersen, Torill Sauer, Jürgen Geisler

**Affiliations:** ^1^ Department of Oncology, Akershus University Hospital (AHUS), Lørenskog, Norway; ^2^ Institute of Clinical Medicine, Faculty of Medicine, University of Oslo, Oslo, Norway; ^3^ Department of Pathology, Oslo University Hospital, Oslo, Norway; ^4^ Department of Clinical Molecular Biology (EpiGen), AHUS, Lørenskog, Norway

**Keywords:** ductal carcinoma *in situ* (DCIS) of the breast, immunohistochemisty, personalized medicine (PM), Ki67 proliferation index, hormone receptors human epidermal growth factor receptor 2 (HER2), International Ki67 in Breast Cancer Working Group (IKWG)

## Abstract

**Simple summary:**

We investigated the role of Ki67, a ubiquitous marker in cancer, within the context of ductal carcinoma *in situ* (DCIS), a precursor of invasive breast cancer. Through rigorous analysis of histopathological and immunopathological samples from a substantial cohort, this study revealed robust correlations between heightened Ki67 expression, diminished progesterone (PR) levels, and HER2 overexpression, indicative of aggressive DCIS phenotypes. These findings offer novel insights into the surrogate immunomolecular subtyping landscape of DCIS, potentially refining risk stratification and therapeutic approaches. This elucidation underscores the translational significance of Ki67 as a prognostic and predictive biomarker in DCIS, with implications for personalized treatment paradigms and patient outcomes.

**Background:**

The Ki67 proliferation index is widely used in various tumors, including invasive breast carcinoma (IBC). However, its prognostic utility is often constrained by technical complexity. Its diagnostic and clinical significance in ductal carcinoma *in situ* (DCIS) remains uncertain. We studied Ki67 immunohistochemistry interobserver diagnostic agreement at different cutoff values in high-grade DCIS. Additionally, we investigated the associations between Ki67 expression, PR levels, and human epidermal growth factor receptor 2 (HER2) in high-grade DCIS among various subtypes (Luminal (Lum) A, LumB HER2^-^, LumB HER2^+^, HER2-enriched, and triple-negative)).

**Methods:**

Using histopathological specimens from 484 patients diagnosed with DCIS between 1996 and 2018, we implemented the 2013 St. Gallen recommendations for surrogate immunomolecular subtyping of IBC. Subtypes were classified, and the Ki67 interobserver diagnostic agreement between Counting Pathologist 1 (CP1) and CP2 was calculated using Cohen’s kappa coefficient at various cutoff values.

**Results:**

The Cohen’s kappa coefficient for interobserver agreement between CP1 and CP2 was κ = 0.586, indicating moderate agreement. Ki67 levels varied significantly among subtypes (*p* < 0.0001), with a median Ki67% being higher in cases with invasive components (*p* = 0.0351). Low PR combined with high Ki67% was significantly associated with HER2 overexpression (*p* = 0.0107).

**Conclusions:**

Interobserver agreement for the Ki67 count was moderate. Ki67 expression showed considerable variability in high-grade DCIS. Low PR levels combined with high Ki67 expression were linked to HER2 overexpression, showing possible clinical implications for identifying high-risk DCIS.

## 1 Introduction

Breast cancer is the second most prevalent form of cancer among women, accounting for over 30% of all newly diagnosed cancer cases in women annually. Forecasts indicate 310,720 new cases and 42,250 deaths among American women in 2024 alone (1, 2). At some point in their lives, approximately 12.5% of women receive a breast cancer diagnosis (1). Currently, there are over four million breast cancer survivors in the United States (1). This includes women who are still being treated and those who have completed the treatment (1). However, this burden extends beyond mere statistics, encompassing a profound impact on patients, the healthcare system, and societal well-being.

The latest data obtained by the Centers for Disease Control and Prevention revealed that breast cancer commands the highest treatment expenditure among all cancers in the United States, with medical services amounting to $26.2 billion and prescription drugs totaling $3.5 billion (3).

Projections anticipate that roughly 56,500 new breast ductal carcinoma *in situ* (DCIS) diagnoses will occur in 2024 (1, 2). Currently, the extent and histological grade of lesions are the only factors that influence DCIS treatment (4). For patients with *in-situ* lesions only, guidelines recommend surgery alone or in combination with radiation for the treatment of patients with *in-situ* lesions only. Despite its prevalence, the precise subtypes of DCIS leading to aggressive cancers remain unidentified, necessitating urgent research. Women diagnosed with DCIS have a 1.8-fold increased risk of breast cancer-related mortality compared to women in general (5). This heightened risk was particularly pronounced among those diagnosed before the age of 35 years, where mortality rates were approximately 17 times higher than expected within nine years following diagnosis (5).

Since the introduction of mammography screening, breast DCIS has become a common diagnosis (6). About 20–25% of all malignant lesions found through national screening programs in industrialized nations are DCIS (6, 7).

In two previous studies, we found that human epidermal growth factor receptor 2 (HER2)-enriched high-grade DCIS was a high-risk subtype because it was strongly linked to having invasive components (8) and low progesterone receptor (PR) levels, correlated with HER2 overexpression and the presence of invasive components (9). The ultimate goal of our research is to improve the identification of DCIS lesions with a high potential to develop into invasive breast carcinoma (IBC). This information may pave the way for personalized treatment for women diagnosed with DCIS. We can potentially evaluate adjuvant immunotherapy (e.g., anti-programmed cell death protein 1/programmed death-ligand 1 (anti-PD-1/PD-L1) therapy) in a subset of patients at the highest risk of developing invasive breast cancer. In addition to enabling patients to avoid under- and over-treatment, changing the current standards can allow the health system to target individuals diagnosed with high-grade DCIS more effectively. In IBC, hormone receptors (HRs) for estrogen and progesterone, in addition to HER2 and Ki67 proliferation index, are all deciding factors according to a complex treatment algorithm (10). Conversely, we have not yet established the utility of immunohistochemistry (IHC) markers in the diagnosis of DCIS. Breast DCIS is a diverse entity, with different growth patterns and nuclear atypia ranging from subtle to severe. Certain DCIS subtypes may not pose an imminent risk, whereas others may suggest the possibility of progression to invasive breast cancer. If left untreated, around 20–25% of DCIS cases progress to invasive breast cancer within ten years (11). In other studies, this proportion has been reported to be as high as 50% (12). The precise DCIS subtypes that progress to aggressive cancers is currently unidentified. However, because almost all women diagnosed with DCIS undergo breast surgery, there is a lack of understanding of the possible consequences of not treating this disease. Despite this, a phase III randomized controlled clinical trial examined the risks and benefits of active monitoring versus standard DCIS therapy in patients with low-risk DCIS. The results of this study have yet to be published (13, 14). Routine IBC specimens frequently contain DCIS components. However, we do not devote much attention to the morphological features of the HR-, HER2-, and Ki67 status of this component, as these factors currently have little bearing on the clinical management of patients with IBC. Only a few studies have investigated the significance of these markers in DCIS according to their molecular subtypes (15–17).

Over the years, Ki67 staining has increasingly permeated cancer research and diagnosis, with applications in numerous malignant tumors, including invasive breast cancer (IBC). The Ki67 proliferation index is a widely acknowledged marker of cellular proliferation, serving diagnostic, prognostic, and predictive roles with notable efficacy. In 1983, Gerdes et al. first discovered Ki67 protein (18). The name was derived from the city of origin (Kiel, Germany), and 67 was the number of original clones in a 96-well plate (19). During all stages of the cell cycle, except for the dormant phase (G0 phase), dividing cells show Ki67 expression in their nuclei (20). Initially, the main purpose of Ki67 was to examine cell cycle and cellular proliferation in normal and malignant tissues. It is now one of the most commonly used markers for determining tumor cell growth and behavior. Ki67 is a feasible marker for predicting the behavior of malignant tumors by quantifying the percentage of actively dividing tumor cells using IHC. Despite its extensive application, research continues to be conducted to improve Ki67 prognostic and predictive accuracy in various cancer types (21).

A large global phase III randomized trial included breast cancer patients with 1-3 positive axillary lymph nodes and a Ki67 index > 20% treated with adjuvant endocrine therapy and abemaciclib, a cyclin-dependent kinase 4 and 6 (CDK4/6) inhibitor. The primary endpoint was invasive disease-free survival. Results showed that while Ki67 was prognostic for recurrence, it was not predictive of response, as abemaciclib provided benefits regardless of Ki67 status (22, 23).

According to the International Ki67 in the Breast Cancer Working Group (IKWG), Ki67 ≤ 5% indicates low proliferation, and Ki67 > 30% indicates high proliferation (24).

Several changes have been made to the Ki67 cutoff values used to identify and differentiate Luminal A (LumA) and LumB subtypes in IBC at St. Gallen consensus meetings. This demonstrates the difficulty in defining low-grade tumors with a low proliferation index versus tumors exhibiting enhanced proliferation (25, 26) which are suitable for therapeutic decisions. In routine diagnosis, the vast majority of IBC cases have an intermediate (6–29%) Ki67 proliferation index (27). Only a limited number of studies (28, 29) have investigated the Ki67 proliferation index in breast DCIS. The purpose of this study was to identify a more consistent cutoff value for Ki67 and investigate whether the Ki67 proliferation index can contribute to identifying high-grade DCIS lesions with the highest potential to finally turn into IBC in humans. Furthermore, we aimed to study the possible relationships between HER2 overexpression, low PR levels, and high Ki67 expression.

## 2 Materials and methods

The present study was a retrospective interventional investigation. Our entire study material included formalin-fixed paraffin-embedded (FFPE) histopathological specimens from a consecutive patient cohort stored in the diagnostic archive at Akershus University Hospital, Norway. Samples from 494 female patients diagnosed with ductal carcinoma *in situ* (DCIS) of the breast between 1996 and 2018 at Akershus University Hospital were collected, examined, and graded by experienced breast pathologists using the Van Nuys classification system (8, 30). The selected patients who met the inclusion criteria (8) received a prepaid return envelope that was enclosed in the letters they received, in addition to a sheet to sign and return if they objected. If they agreed, they would not need to undertake any action. We received reservations from ten patients; consequently, their cases were excluded from further examinations. To our knowledge, this is one of the largest DCIS biobanks in Europe that has been collected and approved for research purposes.

**Figure 1:**
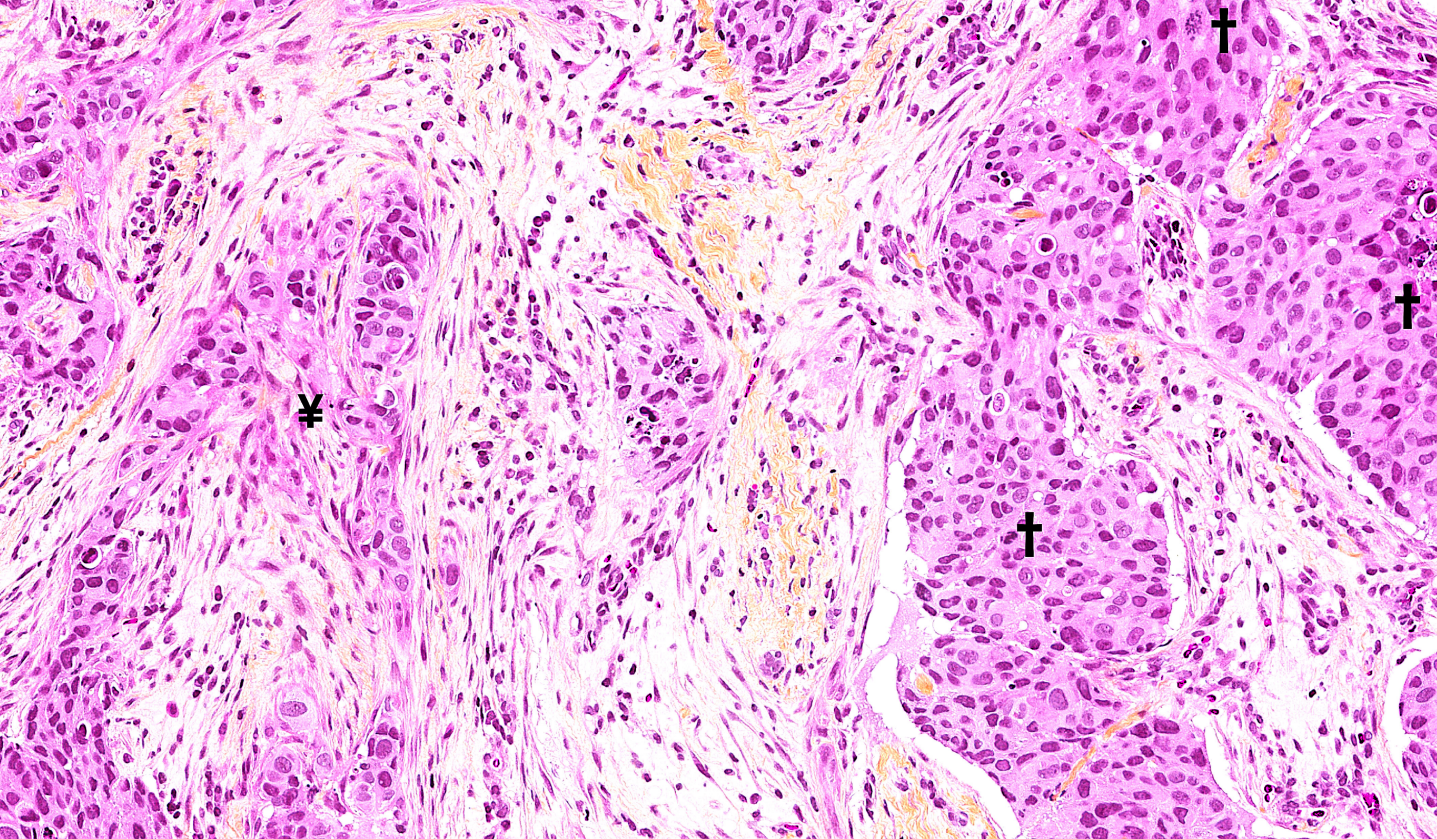
Hematoxylin phloxine saffron-stained sections of the human breast showed retrograde cancerization in the expanded terminal duct lobular unit (TDLU). Focally, it resembled early invasion (¥); however, immunohistochemistry (IHC) staining did not prove or confirm this. A few mitotic figures were observed (†). 20X magnification.

We chose to investigate high-grade (grade 3) DCIS cases because these lesions are believed to harbor the highest risk of recurrence and progression to IBC (30–33). We identified 357 patients with high-grade DCIS (**Figure 1**) and stained their high-grade DCIS tissues using IHC for the estrogen receptor (ER), progesterone receptor (PR), human epidermal growth factor receptor 2 (HER2), and Ki67 proliferation index. ER and PR IHC positivity was defined as ≥ 1% positive DCIS cells, in accordance with the updated guidelines of the American Society of Clinical Oncology (ASCO) and the College of American Pathologists (CAP) (34) developed for IBC.

### 2.1 2013 St. Gallen international consensus conference

We classified each DCIS case into respective subtypes according to the 2013 St. Gallen International Consensus Conference. This classification is used for molecular subtyping of IBC (8, 26). In summary, according to this classification (26), the LumA subtype was defined when ER was positive (≥ 1%) and/or PR-positive (≥ 20%), HER2^-^, and Ki67 index was < 20%. LumB HER2^-^ was defined as ER that was positive, HER2^-^, and Ki67 expression ≥ 20%, or when ER was positive, Ki67 ≥ 20% or PR expression < 20%, and HER2^-^. LumB HER2^+^ was defined when ER and/or PR were positive and HER2^+^ and Ki67 at any value. HER2-enriched was defined as having ER and PR negativity, HER2^+^, and any Ki67 value. Triple-negative (TPN) was defined when ER, PR, and HER2 were negative and Ki67 was at any value. We described the procedures in detail in our previous study (8). General definitions of the molecular subtypes of IBC according to IHC surrogate markers are provided in **Supplementary Table 1** (35, 36).

### 2.2 Ki67 proliferation index ratio

The Ki67 proliferation index was calculated by counting 200 DCIS cells in two separate hotspot foci on IHC-stained slides. The percentage of Ki67-positive cells was recorded as a continuous value rather than as a categorical value. The cutoff threshold for a high Ki67 proliferation index was set at 20%, in accordance with the 2013 St. Gallen recommendations. Three breast pathologists interpreted the primary IHC analyses for ER, PR, HER2, and Ki67, and these results were subjected to further statistical calculations. To determine Ki67 interobserver diagnostic agreement, two additional experienced breast pathologists; Counting Pathologist 1 (CP1) and Counting Pathologist 2 (CP2), recounted 50 DCIS samples each by calculating the ratios of 200 DCIS cells in two separate hotspot foci.

### 2.3 Ki67 interobserver diagnostic agreement

#### 2.3.1 2013 St. Gallen and 2021 IKWG

Interobserver diagnostic agreement was measured using Cohen’s kappa coefficient method at a cutoff value of 20% (2013 St. Gallen recommendations) and < 1–5%, > 5–29%, and ≥ 30% intervals (2021 IKWG recommendations). We also examined the proportion of Ki67 proliferation index in the < 1–5%, > 5–29%, and ≥ 30% intervals in our entire cohort. We combined LumA and LumB HER2^-^ to obtain a sufficiently large sample size and compared the Ki67 distributions in these two combined subtypes with the LumB HER2^+^, HER2-enriched, and TPN subtypes, respectively.

### 2.4 “Pure” and “W/invasive” subcategories

Moreover, we sorted each DCIS subtype into three subcategories: “Pure” (n = 306), meaning those without an invasive component; “W/invasive” (n = 51), meaning those with an invasive component; and “All” (n = 357), meaning the entire group of the given subtype, and the proportions were calculated. We described details regarding the extent of DCIS lesions in a previous study (8).

### 2.5 Non-Ki67 dependent subtypes

We also combined cases belonging to the DCIS subtypes whose classification was not dependent on Ki67 expression, namely the LumB HER2^+^, HER2-enriched, and TPN groups, and compared the proportions of Ki67 index expression in the “Pure” with the “W/invasive” subcategories.

### 2.6 Low PR and high Ki67 combined subcategories

Finally, we combined low PR levels (< 20%) with high Ki67 expression (≥ 20%) and studied the distribution of this combination in LumB HER2^-^, LumB HER2^+^, HER2-enriched, and TPN subtypes.

### 2.7 Immunohistochemistry

A Dako Autostainer (Agilent) was used to perform IHC staining for ER, PR, HER2, and Ki67. Antigen retrieval was achieved in a PT-Link station by immersion in EnVision™ FLEX Target Retrieval Solution at a high pH (K8004, Agilent) and heating at 97°C for 20 minutes. Endogenous peroxidase activity was quenched by incubating the slides in the EnVision™ FLEX peroxidase blocking reagent (K8000, Agilent) for 5 minutes. For HER2 IHC, non-specific staining was inhibited using an animal-free blocking solution 1x (#15019) for 30 minutes. Primary antibodies Ki67 (1:200), ER (1:50), and PR (1:100) were diluted in EnVision™ FLEX Antibody Diluent (K8006, Agilent); antibody HER2 (1:200) was diluted in SignalStain^®^ Antibody Diluent (#8112, Cell Signaling), and slides were incubated with primary antibodies for 20–60 minutes at room temperature. For ER and PR IHC, rabbit (K800921-2, Agilent) and mouse (K800221-2, Agilent) linkers were added for 15 minutes for signal amplification after incubation with the primary antibody. This was followed by incubation with a ready-to-use secondary buffered solution (k8002, EnVision FLEX/HRP, Agilent) for 20 minutes. The sections were reacted with 3,3′-Diaminobenzidine tetrahydrochloride (DAB) solution for 10 minutes. Counterstain with Hematoxylin (link) (k8008, Agilent) for 5 minutes. In each run, a positive tissue control with invasive mammary carcinoma was included. EnVision FLEX/HRP, Agilent) for 20 minutes. In each run, a positive tissue control with invasive mammary carcinoma was included. **Supplementary Table 2** shows details of the antibody clones, staining, and dilutions.

HER2 IHC was scored based on ASCO/CAP guidelines, as in routine diagnostics for IBC (37, 38). Briefly, HER2 was scored “0” (ultra-low) when IHC staining was absent or membranous staining was weak and pale in ≤ 10% of the DCIS cells. HER2 was considered “1+” (low) when partial and incomplete membrane staining also showed a faint intensity within > 10% of the DCIS cells, and HER2 that was scored as “3+” showed strong and complete positive membrane staining in > 10% of the DCIS cells.

### 2.8 Dual-color silver-enhanced in situ hybridization

HER2 was recognized as “2+” (low) when the membrane was faint to moderate in > 10% of DCIS cells, which was considered equivocal, and was subjected to further dual-color silver-enhanced *in situ* hybridization (dc-SISH) analysis performed on a Ventana BenchMark Roche Diagnostics machine using the fully automated Ultra-IHC/ISH Staining Module (39) with CC2 as a buffer. The dc-SISH results were interpreted in accordance with the updated ASCO/CAP comprehensive guidelines and algorithms established for IBC (40). We described the details of dc-SISH assessments in our cohort in a previous publication (9). Regardless of when the sample was collected, we did not observe any changes in the quality or intensity of ER, PR, Ki67, HER2 IHC, or HER2 dc-SISH.

### 2.9 Statistical analysis

We used GraphPad Prism version 10.2.1 for the statistical calculations. We applied different tests to calculate statistical significance. The Mann-Whitney non-parametric test was used to compare variables between the two independent groups. The Kruskal-Wallis non-parametric test was used to compare the independent measurements of variables between multiple subtypes. Pearson’s chi-squared (χ²) or Fisher’s exact tests were used to calculate *p*-values when comparing two proportions using the contingency table. The confidence interval in the odds ratio (OR) was calculated using the Baptista-Pike test in contingency tables. We calculated Cohen’s kappa coefficient for interobserver agreement (41) using the online calculator provided by GraphPad Prism (42). Statistical significance was set *a priori* at *p* < 0.05.

## 3 Results

### 3.1 Ki67 interobserver diagnostic agreement

In our cohort of 357 patients with high-grade ductal carcinoma *in situ* (DCIS), by applying the 2021 IKWG recommendations, we identified 81 cases (23%) with a Ki67 value between a < 1–5% interval, while 49 (14%) cases had a value above 30%. The majority of the Ki67 values (n = 227; 64%) were in the > 5–29% interval. The mean and median Ki67 indices in the selected 50 cases that Counting by Pathologist 1 (CP1) and CP2 assessed were 23% and 22%, respectively. In these 50 cases, we found differences in the Ki67 scores between CP1 and CP1, with mean (21% and 25%), median (20% and 25%), minimum (2% and 4%), and maximum (44% and 65%) values, respectively. Cohen’s kappa coefficient for interobserver agreement between CP1 and CP2 was κ = 0.586 (95% confidence interval (CI): 0.361 to 0.812), indicating moderate agreement when we used the 20% cutoff (2013 St. Gallen). We then applied the 2021 IKWG recommendations and calculated the Cohen’s kappa coefficient in > 5–29% and ≥ 30% intervals and found κ = 0.279 (CI: −0.006 to 0.564) and κ = 0.221 (CI: −0.067 to 0.508), respectively, indicating fair agreement in both of these intervals. The calculated Cohen’s kappa coefficient in < 1–5% was κ = 1.000. CP1 scored below the 20% Ki67 cutoff value in 6 of 50 cases (12%), whereas CP2 scored above it. In contrast, CP1 scored above this cutoff in 1 of 50 cases (2%), whereas CP2 scored below it.

**Figure 2:**
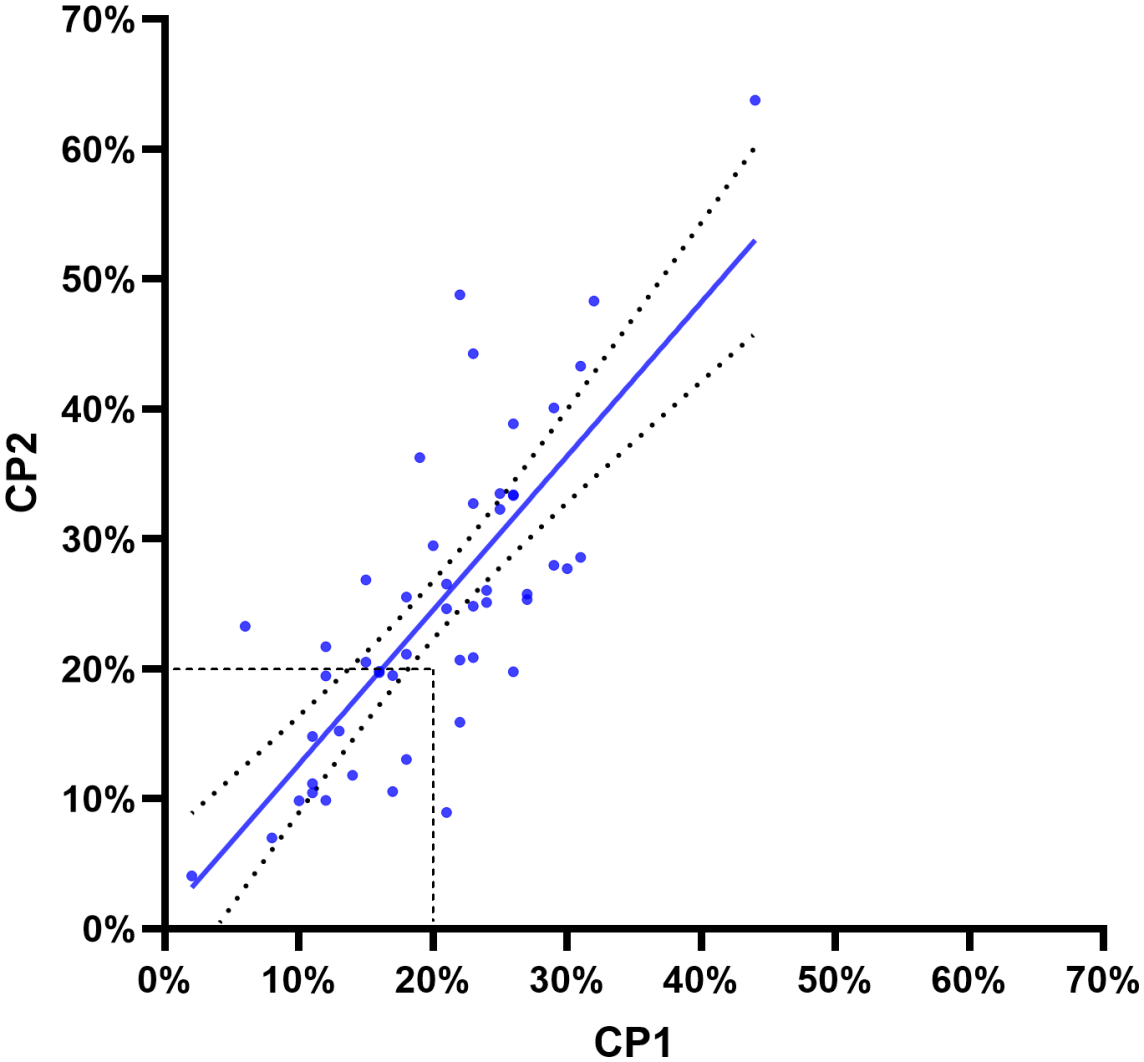
The Pearson correlation coefficient (*r*) was 0.7667, with a two-tailed p < 0.001. R^2^ was 0.5878 (95% confidence interval: 0.6208–0.8612). The two curved lines show the 95% confidence bands. Dashed lines show a cutoff of 20% according to the 2013 St. Gallen guidelines for invasive breast carcinoma. The line of best fit was derived using least-squares regression.

The observed agreement ratio was 80%, whereas the expected agreement ratio by chance was 51.7%, as shown in **Figure 2**.

### 3.2 Ki67 distribution across subtypes

**Figure 3:**
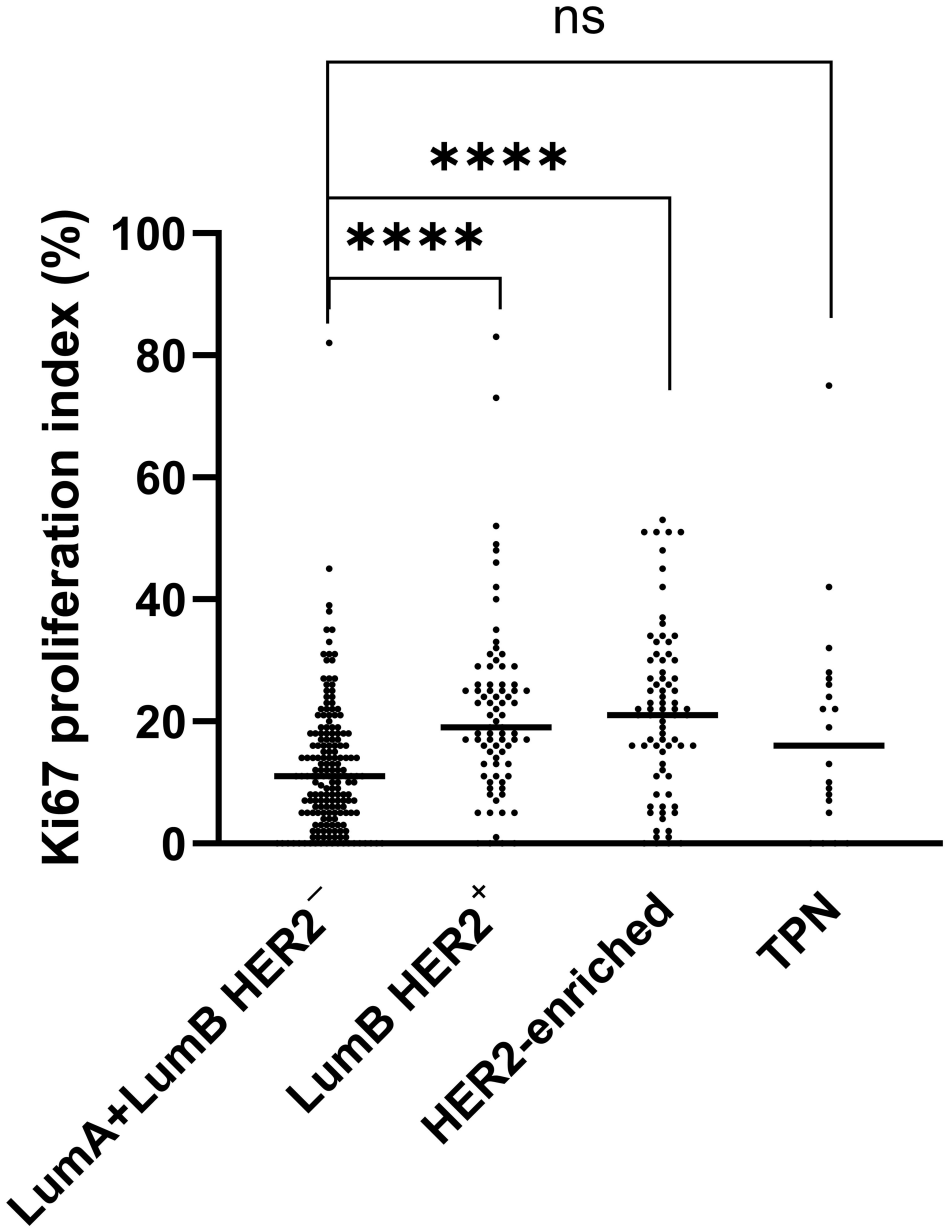
Significant differences (*p* < 0.0001) are marked with (****) using the Kruskal-Wallis test. A scatter plot with median values indicates a comparison of Ki67 expression. Luminal (Lum) A was combined with LumB HER2^-^ into one group and compared with LumB HER2^+^, HER2-enriched, and TPN subtypes, respectively.

**Figure 4:**
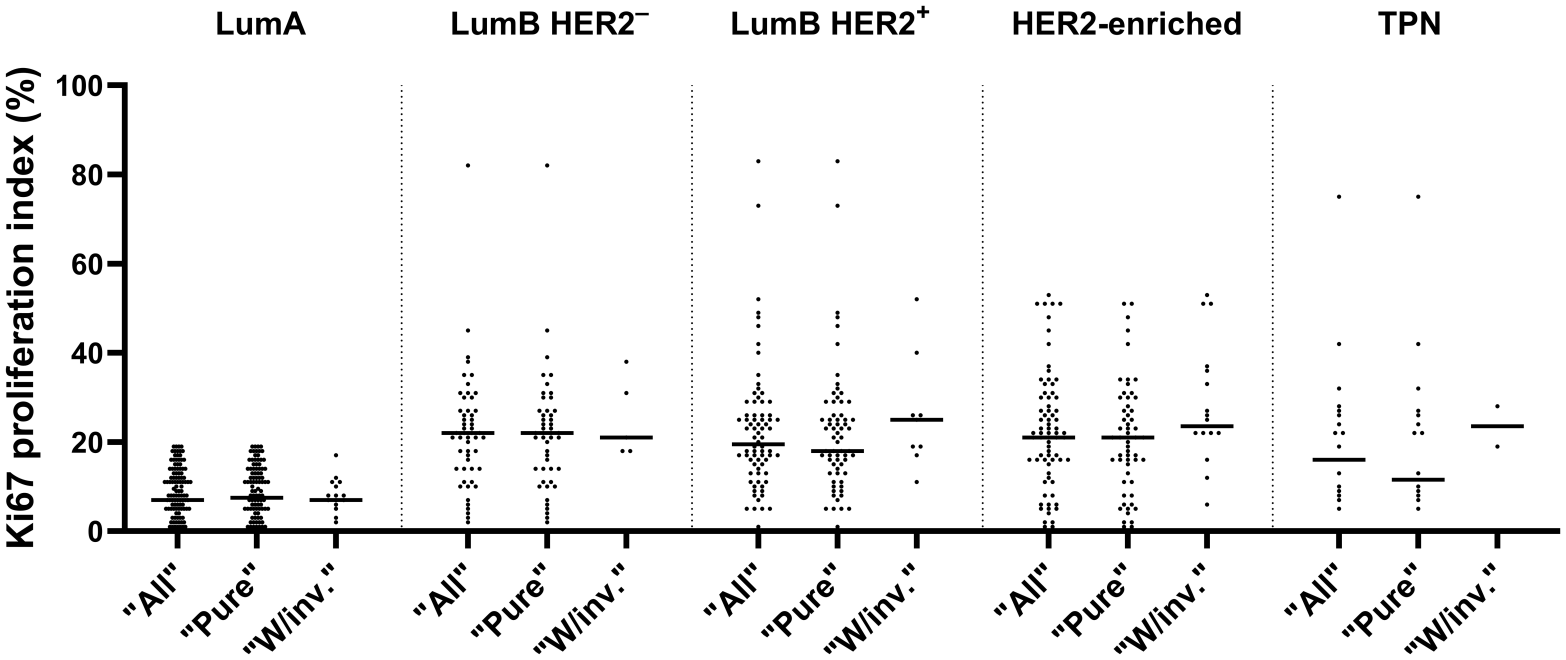
Distribution of Ki67 in various subtypes, subcategorized as “All”, “Pure” and “W/invasive” is shown with median values in a scatter plot.

We found significant differences in the distribution of Ki67 (p-adjusted < 0.0001, Kruskal-Wallis test) between the combined LumA and LumB HER2^-^ subtypes cases when we compared them to the LumB HER2^+^ and HER2-enriched subtypes cases (**Figure 3**). Remarkably, the comparison with the TPN subtype did not show a significant difference (p-adjusted = 0.5886, Kruskal-Wallis test), as shown in **Figure 3**. Notably, the Ki67 proliferation index varied greatly (range < 1% and 83%, median 21%) among subtypes, of which their classifications were not dependent on Ki67 expression (LumB HER2^+^, HER2-enriched, and TPN) (**Figure 3**). We also looked into the proportions of Ki67 subcategorized in “Pure,” “W/invasive,” and “All” in every subtype. **Figure 4** shows the Ki67 proliferation index for each case placed in the appropriate DCIS subtype and subcategory. **Table 1** summarizes the Ki67 mean, median, and range values for each subtype and subcategory.

**Table 1:** Details about the Ki67 expression values (minimum, maximum, mean and median), subcategorized in “Pure” meaning those without an invasive component; “W/invasive” meaning those with an invasive component, “All” meaning the entire group of the given subtype.

Subtype	Min. (%)	Max. (%)	Mean (%)	Median (%)
LumA“All”n = 127	<1	19	8	7
“Pure”n = 110	<1	19	8	7
“W/invasive”n = 17	<1	17	7	7
LumB HER2^-^“All”n = 53	2	82	22	22
“Pure”n = 48	2	82	22	22
“W/invasive”n = 5	18	38	25	21
LumB HER2^+^“All”n = 79	<1	83	22	20
“Pure”n = 70	<1	83	21	18
“W/invasive”n = 9	11	52	26	25
HER2-enriched“All”n = 78	<1	53	21	21
“Pure”n = 60	<1	51	20	21
“W/invasive”n = 18	<1	53	26	24
TPN“All”n = 20	<1	75	18	16
“Pure”n = 18	<1	75	18	12
“W/invasive”n = 2	19	28	24	24
All cases “W/invasive”n = 51	<1	53	20	18

### 3.3 Ki67 expression in non-Ki67 dependent DCIS subtypes

**Figure 5:**
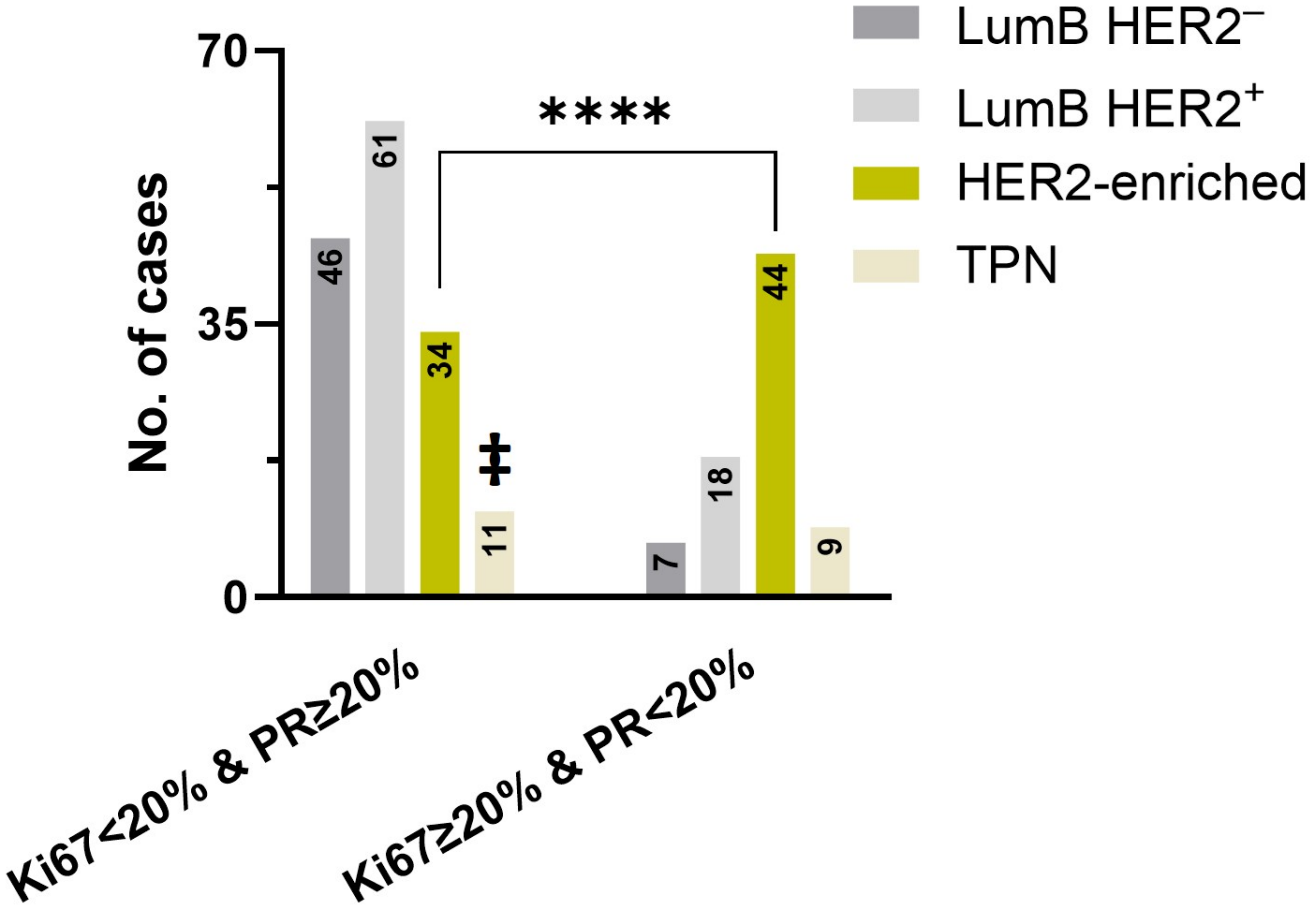
A significant difference (*p* < 0.0001) was found in the HER2-enriched subtype, as indicated by the asterisk (****) using the chi-squared test. The number of cases with Ki67 < 20% & PR ≥ 20% (left) and Ki67 ≥ 20% & PR < 20% (right) subtypes was compared between LumB HER2^ˉ^, LumB HER2^+^, HER2-enriched, and TPN, respectively. ‡ indicates that TPN cases on the left expressed only Ki67 < 20% and were PR-negative.

We next examined the Ki67 expression in combined DCIS subtypes whose classification was not dependent on Ki67 (LumB HER2^+^, HER2-enriched, and TPN) and found statistically significant higher Ki67 expression in “W/invasive” compared to “Pure” subcategories (*p*-exact = 0.0351, Mann-Whitney test). In this combined subcategory, the median Ki67 expression was 25% (n = 29) in “W/invasive” and 19% in “Pure” (n = 148). We chose to identify patients with low PR levels (< 20%) in combination with a high Ki67 expression (≥ 20%) phenotype, in accordance with the cutoff values determined at the 2013 St. Gallen Consensus Conference. A total of 78 of the 357 patients (22%) fulfilled this criterion. Of these, seven cases (9%) were LumB HER2^-^, 18 cases (23%) were LumB HER2^+^, 44 were HER2-enriched (56%), and nine cases belonged to the TPN subtype (12%). The HER2-enriched subtype was significantly overrepresented (p < 0.0001, chi-squared test) (**Figure 5**).

### 3.4 Comparison of high Ki67, low PR levels, and HER2 expression score

**Figure 6:**
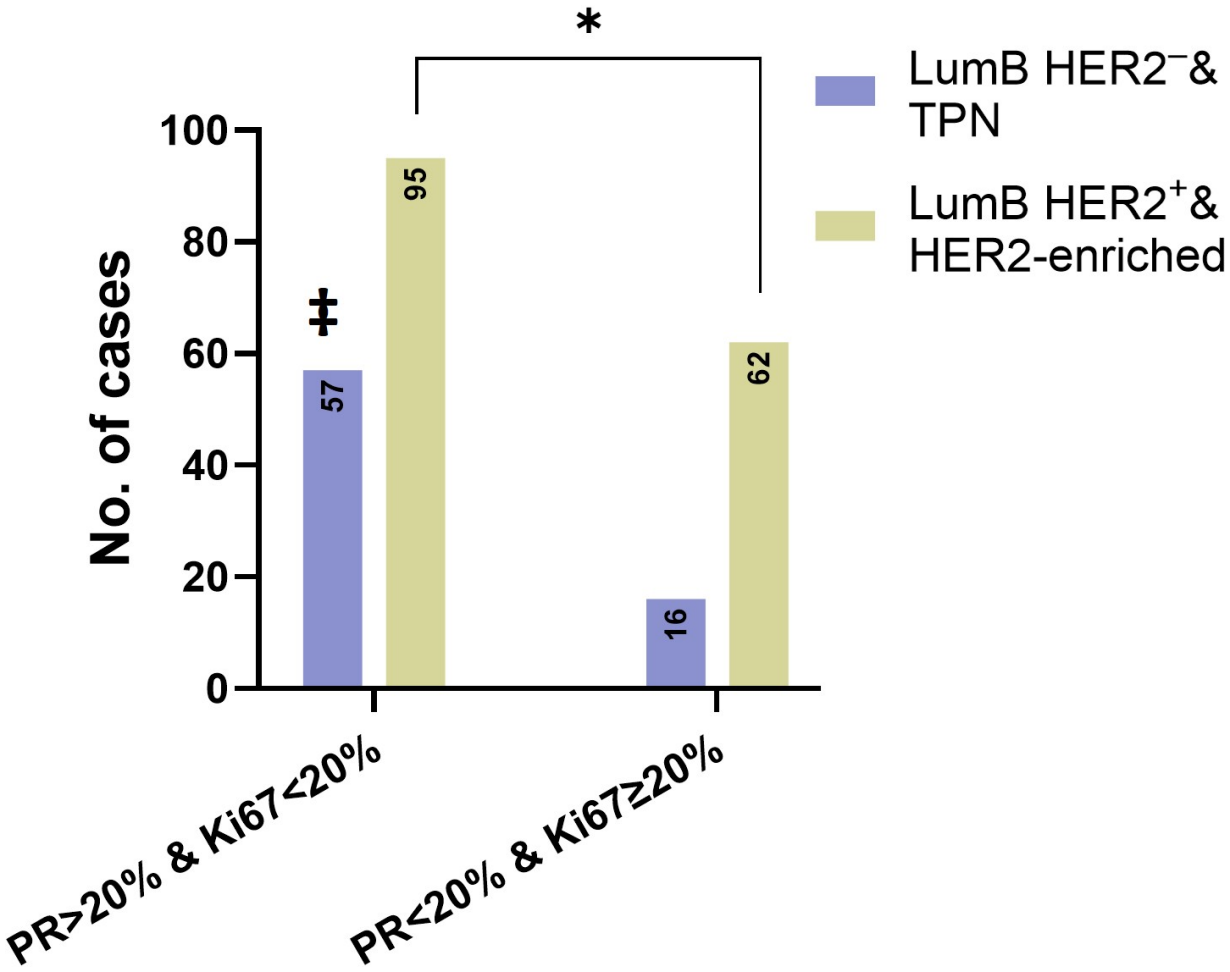
The asterisk (*) and Fisher’s exact test showed that there was a significant difference (*p* = 0.0107) between the combined LumB HER2^ˉ^ and TPN subcategory and combined LumB HER2^+^ and HER2-enriched subcategory. The number of cases with Ki67 < 20% & PR ≥ 20% (left) and Ki67 ≥ 20% & PR < 20% (right). ‡ indicates that TPN cases on the left expressed only Ki67 < 20% and were PR-negative.

We also compared the number of patients with a combined low PR level (< 20%) and high Ki67 expression (≥ 20%) phenotype (2013 St. Gallen) among cases with HER2 overexpression (score 3+), low (score 1+) and ultra-low (score 0) HER2 expression. Out of 78 cases with low PR and a high Ki67 phenotype, 62 belonged to the combined LumB HER2^+^ and HER2-enriched subcategories, which were significantly higher compared to the combined LumB HER2^-^ and TPN subcategories (p = 0.0107, Fisher’s exact test) (**Figure 6**).

## 4 Discussion

In this study, we identified that the interobserver agreement between Counting by Pathologist 1 (CP1) and CP2 varied with the selection of Ki67 cutoff values. Cohen’s kappa coefficient was higher, and the interobserver agreement between CP1 and CP2 was more reproducible in our assessed 50 cases when we used the 20% cutoff (moderate), compared to > 5–29% and ≥ 30% intervals (fair). The average mean and median Ki67 expression at the 20% cutoff value in the 50 assessed cases, scored by CP1 and CP2, were 20.5% and 23%, respectively, which could be a possible explanation for the higher reproducibility in favor of the 20% cutoff value. Nonetheless, these 50 cases were probably not large enough to cover the entire Ki67 expression spectrum in high-grade DCIS. For instance, the calculated Cohen’s kappa coefficient κ = 1.000 was unreliable because only one case was found to have a Ki67% of ≤ 5%. Poor, fair, and moderate Ki67 interobserver agreements have also been found in studies involving IBC cases (43–45).

In our 357 high-grade DCIS cohort, the majority of patients (64%) had a Ki67 value in a > 5–29% interval. This is in accordance with the findings in IBC (27). Harbeck et al. performed a large prospective phase III trial on endocrine therapy in IBC. They found that Ki67 levels ≥ 20%, along with other high-risk factors, could be used to identify patients who might benefit from certain treatments (46). An important issue in the analytical phase of Ki67 IHC assessment was the use of multiple counting methods (47–49).

We acknowledge that DCIS, classified as the LumA and LumB HER2^-^ subtypes, share several molecular features. The HRs are positive, and HER2 is negative in both subtypes. These two subtypes are defined and distinguished by an arbitrary and predefined Ki67 cutoff value, which has undergone several changes over the past decade. Thus, the Ki67 measurements, both mean, median, and maximum, should be interpreted cautiously in these two subtypes. We chose to combine these two subtypes to obtain a statistically large number of patients.

We demonstrated a statistically significant higher Ki67 distribution in LumB HER2^+^ and HER2-enriched subtypes (*p*-adjusted < 0.0001, Kruskal-Wallis test) when these subtypes were compared to the combined LumA and LumB HER2^-^ subtypes as an entire group. Remarkably, the comparison with the TPN subtype was not significant (*p*-adjusted = 0.5886, Kruskal-Wallis test) (**Figure 3**).

The results of this study indicated that high-grade DCIS cases with low PR levels (< 20%) combined with high Ki67 expression (≥ 20%) were more likely to be of the HER2-enriched subtype (*p* < 0.0001, chi-squared test) (**Figure 5**). By definition, the HER2-enriched subtype of invasive cancer is a non-dependent Ki67 entity, defined by the HER2 score 3+ and simultaneous negative HRs, irrespective of whether Ki67 is low or high. Despite this, DCIS cases with low PR levels (< 1%) combined with a high Ki67 expression constituted a strong majority of cases in this subtype (**Figure 5**). Additionally, cases with low PR levels and simultaneously high Ki67 expression were significantly overrepresented among the combined group of LumB HER2^+^ and HER2-enriched subtypes, representing 79% (62 out of 78) of the cases (*p* = 0.0107, Fisher’s exact test) (**Figure 6**). We suggest that, in a given high-grade DCIS lesion with a low PR level and high Ki67 expression, there is a 56% probability that the lesion is a HER2-enriched subtype and a 79% probability of HER2 overexpression (score 3+) (OR: 2.325; 95% CI: 1.229 to 4.518). In an earlier study, we found that the HER2-enriched subtype was significantly associated with invasive components (8). We believe that these findings can potentially identify high-risk DCIS patients.

Interobserver variations in Ki67 visual scoring are particularly evident in the intermediate interval (> 5% and < 30%) (24). As a result, in a recent guideline by the IKWG in 2021, Ki67 cutoff values of ≤ 5% or ≥ 30% were preferred for use in IBC. Ki67 clinical utility should be limited to the estimation of prognosis in anatomically favorable ER-positive and HER2^-^ patients and to determining which patients do not require adjuvant chemotherapy (24).

In 2008, the Breast International Group Trial 1-98 (BIG-1-98), set the cutoff value to define a high Ki67 proliferation index in IBC at 11% (50). The IKWG was established in 2009 to address preanalytical, analytical, and interlaboratory challenges regarding Ki67 (51, 52). Later, at the 2011 St. Gallen International Consensus Conference, a cutoff threshold of 14% was established (25), to distinguish between the LumA and LumB HER2^-^ subtypes of IBC. Two years later, at the 2013 St. Gallen meeting, the threshold for a high Ki67 proliferation index was increased to 20% following discussions on the poor repeatability of the previous threshold values (26).

Nonetheless, the 2015 St. Gallen Conference did not attempt to determine a certain threshold for Ki67 but rather addressed that to be decided based on local laboratory guidelines and routines (53). During the preanalytical phase, multiple variables could have influenced the Ki67 evaluation. These included the type of specimen, type of fixative, length of fixation, and cold ischemic time (the interval from the time the specimen was removed during surgery to placement for tissue fixation) (54).

Compared with ER or HER2 IHC, Ki67 IHC seems to be more sensitive to fixation-related changes. When using fixatives other than neutral buffered formalin, delays of more than 3 hours (too short), 16 hours, or 14 days (too long) in fixation duration may result in a decline in the Ki67 index values. Prolonged fixation periods can potentially cause epitope degradation (55). Ki67 is also more vulnerable to antigen decay during long-term storage in FFPE blocks (56). The IKWG advises performing Ki67 IHC within five years of tissue placement in these blocks (24).

Overall, we deemed the quality of hematoxylin and eosin (HE) morphology and IHC staining in our materials to be technically adequate. In our previous study (8, 57), we experienced that suboptimal fixation was the likely cause of IHC failure in 12 of 422 samples. Nevertheless, we cannot rule out that some FFPE blocks could have poor quality for Ki67 IHC staining (cf. ratios < 1%), even though the ER, PR, and HER2 IHC markers showed acceptable features.

**Figure 7:**
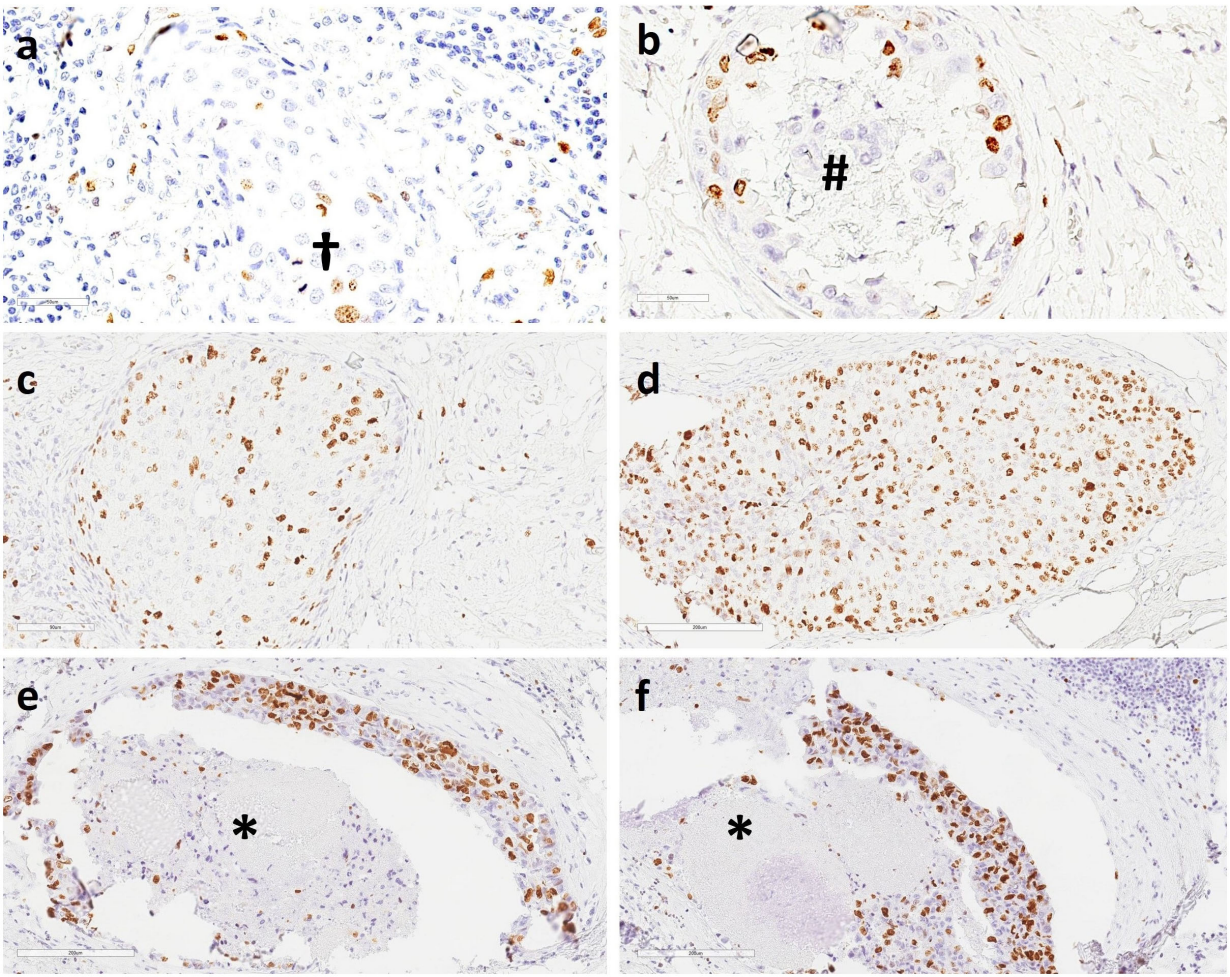
Immunohistochemistry (IHC) staining of DCIS showed **(A)** scant Ki67-positive nuclei in retrograde cancerization in the terminal duct lobular unit (TDLU) and two mitotic figures (**†**); **(B)** a slightly increased number of Ki67-positive nuclei; calcification is seen in the lumen of a mammary milk duct (**#**); **(C)** a moderate number of Ki67-positive nuclei in retrograde cancerization in the TDLU; **(D)** a high number of Ki67-positive nuclei; **(E–F)** a high number of Ki67-positive nuclei in mammary milk ducts. Necrotic cell debris is present in the duct lumen (*). 20X magnification.

In our cohort (n = 357), we chose the hotspot method (**Figures 7A–F**) and calculated Ki67 ratios as a continuous variable rather than a categorical variable in accordance with the current local and national guidelines for the diagnosis, treatment, and follow-up of patients with IBC (58). The IKWG recently recommended global (average) counting instead of hotspot counting, which counts only the areas with the highest proliferative activity owing to its association with higher interobserver variability. In general, there are no established guidelines on how and where to count Ki67 in DCIS or where to expect to find hotspots.

In the assessment of IBC sections, Ki67-positive tumor cells are usually counted on the periphery of invasive lesions (invasion front). The central areas of IBC may be devoid of mitosis and Ki67-positive cells. In DCIS, there may exist a growth front with more pronounced proliferation on the periphery of the DCIS and possibly also a retrograde growth into the lobules (cancerization of lobules).

In addition, growth occurs in the basal layer of DCIS. We selected IHC sections from among those with the largest areas of DCIS, which mostly corresponded to the central areas of the lesions and hence may show poor mitotic figures. These included foci of lobular cancerization. Indeed, we found and counted hotspots in many lobules. Growth was observed in the basal cell layer of the central ducts (28), where the expression pattern varied from low to high.

Digital image analysis (DIA) platforms that can detect nuclear IHC biomarkers, such as Ki67, have made biomarker evaluations faster and easier to perform automatically (59). Thus, it is likely that future Ki67 counting precision, repeatability, and reproducibility will improve, as discussed by other researchers (21, 60, 61). Ki67 should not be widely used for clinical management without evaluation of quality assurance (EQA) because regular participation in such programs has been shown to substantially improve interlaboratory consistency (24, 62).

Our results emphasize the elusiveness of Ki67 in determining a robust and reproducible cutoff. Nevertheless, Ki67 is a valuable prognostic and predictive marker, although it has certain limitations (63–65).

### 4.1 Strengths of the study

To the best of our knowledge, this is the first study to demonstrate a robust correlation between elevated Ki67 expression, low PR levels, and HER2 overexpression, which are indicative of aggressive DCIS phenotypes. We also examined the interobserver agreement between pathologists when counting Ki67 expression in high-grade DCIS, which is a novel study. Furthermore, our cohort consisted of a substantial number of DCIS samples (n = 494) collected over a 22-year period. Qualified mammary pathologists assessed and rated all the samples. We used nationally standardized methods and performed all IHC analyses in a single laboratory.

### 4.2 Limitations of the study

The number of assessed samples (n = 50) regarding the interobserver agreement between pathologists when counting Ki67 expression values was likely not large enough to represent the continuous expression of Ki67 scores. Moreover, we did not have access to patients’ clinical follow-up data in this study.

## 5 Conclusions

The Ki67 interobserver agreement was moderate at a cutoff value of 20%. We demonstrated that patients with invasive components had considerably higher Ki67 values. Ki67 was substantially higher in the LumB HER2^+^ and HER2-enriched subtypes compared to the LumA and LumB HER2^-^ subtypes as a combined group. High Ki67 expression in high-grade DCIS can indicate potential invasive capabilities. Patients with HER2 overexpression (score 3+) were strongly overrepresented when the subtypes were subcategorized by combining a low PR level with a high Ki67 ratio. We believe that this combination has the potential to serve as a prognostic biomarker for invasive growth in high-grade DCIS. We also believe that this study contributes to the identification of DCIS lesions that ultimately progress to IBC and require tailored therapies.

## Data Availability

The original contributions presented in the study are included in the article/**Supplementary Material**. Further inquiries can be directed to the corresponding author.
