# Supplementary material for: High Ki67 expression, HER2 overexpression, and low progesterone receptor levels in high-grade DCIS: significant associations with clinical practice implications
Source: Front Oncol. 2025 Jan 28;15:1467664. doi: 10.3389/fonc.2025.1467664 (PMC11826238; doi:10.3389/fonc.2025.1467664)
Supplement: Supplementary file 1 [file Table1.docx]

**Supplementary Table 1.** **Definition of molecular subtypes for invasive breast carcinoma, according to surrogate immunohistochemistry markers**

| Luminal A | Hormone receptor (ER and/or PR) positive, HER2ˉ, a low level of proliferation according to the Ki67 proliferation index. Luminal A tumors are low-grade, tend to grow slowly, and have the best prognosis. |
| --- | --- |
| Luminal B HER2ˉ | Hormone receptor (ER and/or PR) positive, HER2ˉ, and with higher levels of Ki67 proliferation index, than Luminal A tumors. |
| Luminal B HER2^+^ | Hormone receptor (ER and/or PR) positive, HER2+, and any value level of Ki67 proliferation index. |
| HER2-enriched | Hormone receptor (ER and PR) negative, HER2+, and any value level of Ki67 proliferation index. |

**Supplementary Table 2.** **Details about clone, staining, ID, vendor and dilution of antibodies used in this study**

| Antibody | Clone | Staining | Reference ID | Vendor | Dilution |
| --- | --- | --- | --- | --- | --- |
| Anti-Ki67 | MIB-1 | Nuclear | M724001-2 | Agilent | 1:200 |
| Anti-Human  Estrogen  Receptor α | EP1 | Nuclear | M364301-2 | Agilent | 1:50 |
| Anti-Human Progesterone Receptor | PR 636 | Nuclear | M356901-2 | Agilent | 1:100 |
| Anti-HER2/ErbB2 | D8F12 | Membranous | #4290 | Cell Signaling | 1:200 |
